# Gut Microbiota Perturbation in Early Life Could Influence Pediatric Blood Pressure Regulation in a Sex-Dependent Manner in Juvenile Rats

**DOI:** 10.3390/nu15122661

**Published:** 2023-06-07

**Authors:** Yang Yang, Jinxing Li, Zhimo Zhou, Simou Wu, Jincheng Zhao, Wen Jia, Meixun Liu, Xi Shen, Fang He, Ruyue Cheng

**Affiliations:** Department of Nutrition and Food Hygiene, West China School of Public Health and West China Fourth Hospital, Sichuan University, Chengdu 610041, China; yywishyoubehappy@stu.scu.edu.cn (Y.Y.); m17796410493@163.com (J.L.); gwrzzm@163.com (Z.Z.); simonomon@163.com (S.W.); m15221185092@163.com (J.Z.); 15881869162@163.com (W.J.); lmx_0818@163.com (M.L.); hxgwshenxi@sina.com (X.S.); hf18602880124@163.com (F.H.)

**Keywords:** gut microbiota, blood pressure, ceftriaxone, high-fat diet, pediatric hypertension

## Abstract

The present study aimed to investigate whether gut dysbiosis induced by ceftriaxone in early life could influence pediatric blood pressure regulation in childhood with or without exposure to a high-fat diet (HFD). Sixty-three newborn pups of Sprague-Dawley rats were administered ceftriaxone sodium or saline solution until weaning at 3 weeks, and the rats were fed a HFD or regular diet from 3 to 6 weeks. Tail-cuff blood pressure, the expression levels of genes of the renin-angiotensin system (RAS), the concentrations of IL-1β, IL-6, and TNF-α in the colon and prefrontal cortex, and the composition of fecal microbiota were analyzed. Ceftriaxone treatment significantly increased the diastolic blood pressure of male rats at 3 weeks. At 6 weeks, systolic blood pressure (SBP) was significantly increased only in ceftriaxone treated male rats fed with HFD. The RAS showed increased activation in the kidney, heart, hypothalamus, and thoracic and abdominal aorta of male rats, but only in the kidney, heart, and hypothalamus of female rats. HFD-fed female rats showed a decreased level of IL-6 in the colon. α diversity of gut microbiota decreased and the Firmicutes to Bacteroidetes ratio increased in both male and female rats at 3 weeks; however, these parameters recovered to various degrees in female rats at 6 weeks. These results revealed that early-life gut dysbiosis induced by antibiotics combined with a HFD in childhood could be involved in pediatric blood pressure regulation and an increase in SBP in juvenile rats, and these effects occurred in a sex-dependent manner.

## 1. Introduction

Hypertension is a global public health concern. In addition to the nonmodifiable genetic predisposition, obesity is well known as one of the risk factors of hypertension, together with high salt and low potassium intake [1]. Emerging evidence shows that gut microbiota also plays an important role in blood pressure regulation. Yano et al. [2] reviewed the differences in gut microbiome composition among persons with or without hypertension, where they stated that the loss of biodiversity in the gut microbiome, the reduced short-chain-fatty-acids (SCFAs)-producing gut bacteria, and the increased lipopoly-saccharide-producing gut bacteria may increase individuals’ susceptibility to developing hyper-tension. In recent years, chronic noncommunicable diseases are being increasingly diagnosed in young people, and pediatric hypertension has become a prevalent condition. Previous studies have indicated that pediatric hypertension may persist till adulthood [3]. It, however, remains unclear whether and how gut microbiota is involved in the onset of pediatric hypertension.

Considering the theory of Developmental Origins of Health and Disease, which states that the environment of early life can affect the health in later life and even in adulthood, gut dysbiosis induced by some adverse factors (e.g., antibiotic exposure and a high-fat diet [HFD]) in early life is associated with diseases in later life. Our previous studies demonstrated that early life is the key window for gut microbiota formation and for the development of the intestinal tract, immune system, and brain, and early-life gut dysbiosis could increase the susceptibility of the host to developing colitis, allergic diseases, and neurodevelopmental disorders [4,5,6]. Therefore, we speculate that early-life gut dysbiosis induced by adverse factors, such as antibiotic exposure and HFD, could influence blood pressure regulation during childhood, thus increasing the likelihood of the host to experience hypertension in later life. However, few studies have focused on the relationship between early-life gut microbiota and pediatric hypertension. Moreover, in these studies, most of the animal models used were adults, and the interventions were conducted at birth, while the outcomes were noted in adulthood [7,8]; consequently, the effects of early-life gut microbiota on pediatric hypertension and the underlying mechanisms remain unclear.

In addition to gut microbiota itself, its metabolites may also be involved in blood pressure regulation. SCFAs, mainly produced from high-fiber foods by the gut microbiota, are thought to be involved in maintaining blood pressure balance [9,10,11]. Trimethylamine (TMA), another gut microbiota metabolite, is oxidized to trimethylamine N-oxide (TMAO) by flavin-containing monooxygenases (FMOs) in the liver [12], and TMAO may participate in the progression of diabetes and cardiovascular diseases [13,14]. Components of the immune response, particularly proinflammatory cytokines, also play a role in blood pressure regulation [15]. It is also well known that the renin–angiotensin system (RAS) is an important blood pressure regulatory system in the body. Hsu et al. [7] considered that intestinal microbiota disturbance and barrier function disruption in early life may induce oxidative stress and systemic and neuroinflammation through inflammatory mediators, microbial metabolites, circulating bacteria, and RAS overactivation, thereby leading to the development of hypertension. However, this hypothesis requires confirmation through the study of blood pressure regulation in childhood.

Therefore, the present study aimed to investigate the effects of early-life gut microbiota perturbation induced by antibiotic exposure and HFD on blood pressure regulation in childhood and the potential mechanisms involving the RAS, gut microbial metabolites, and inflammatory responses.

## 2. Materials and Methods

### 2.1. Animal Breeding

The animal experimental protocols were approved by the Ethics Committee of West China School of Public Health and West China Fourth Hospital, Sichuan University (ethical approval code: Gwll2022057). Male and female Sprague-Dawley rats aged 10 weeks were purchased from Beijing Vital River Laboratory Animal Technology Co., Ltd. (Beijing, China), with adaptive feeding for 1 week. After mating and pregnancy, the newborn pups were used for this experiment. During the first 3 weeks of age, ceftriaxone sodium (50 mg/kg body weight [BW]) or saline solution was administered daily to the pups via oral gavage. Male and female pups were raised separately after weaning at 3 weeks of age. All rats were allowed to eat freely a regular diet (purchased from Institute of Laboratory Animals of Sichuan Academy of Medical Sciences and Sichuan Province People’s Hospital, and the components are shown in Table S1) or a HFD (60% kcal% fat, purchased from Research Diets, Inc., New Brunswick, NJ, USA, product number: D12492, and the components are shown in Table S2) until the age of 6 weeks. The rats were then assigned to four groups for this experiment: the saline solution and regular diet treatment group (Control group, *n* = 6, male rats, *n* = 9, female rats); the ceftriaxone sodium and regular diet treatment group (CEF group, *n* = 7, male rats, *n* = 9, female rats); the saline solution and high-fat diet treatment group (HFD group, *n* = 8, both male and female rats); and the ceftriaxone sodium and high-fat diet treatment group (CEF + HFD group, *n* = 7, male rat, *n* = 9, female rats).

### 2.2. Tail-Cuff Pressure Measurement

At the age of 3 and 6 weeks, the systolic blood pressure (SBP) and diastolic blood pressure (DBP) of the rats were measured using the tail-cuff method (BP-600A, Chengdu Techman Software Co., Ltd., Chengdu, China). Briefly, the rats were placed in cages on the machine’s platform, with their tails passing through the tail cuffs. The rats were then allowed to adapt to the tail-cuff inflation. After the rats calmed down, SBP and DBP values were recorded for at least 10 cycles.

### 2.3. Feces Collection, DNA Extraction, and Analysis of Microbiota Composition

At the age of 3 and 6 weeks, feces of each rat were collected in sterilized centrifuge tubes and immediately frozen at −80 °C. For samples of rats at 3 weeks of age, 6 mixed samples of 100 mg in each group were prepared. At 6 weeks of age, samples of 100 mg from each rat were prepared. Fecal DNA was isolated using the TIANamp stool DNA kit (Tiangen Biotech (Beijing) Co., Ltd., Beijing, China) and stored at −80 °C. 16S rRNA gene sequencing was performed by Beijing Novogene Co., Ltd. (Beijing, China). Briefly, after genomic DNA extraction, amplicon generation by polymerase chain reaction (PCR), and the quantification and identification of the PCR products, the library was sequenced using an Illumina NovaSeq platform. Next, the data were fragmented and filtered, and the chimera sequences were then removed to obtain the effective tags. Denoising was then performed with DADA2 or deblur module in QIIME2 software (version QIIME2-202006) to obtain initial amplicon sequence variants (ASVs). The normalized data of the absolute abundance of ASVs were used for the subsequent analysis. α diversity, principal coordinate analysis (PCoA), histograms, and heatmaps of the relative abundance of species were processed and analyzed using the Novomagic, a free online platform for data analysis (https://magic.novogene.com, accessed on 29 September 2022).

### 2.4. Euthanasia of Rats and Organ Collection

Rats were euthanized by anesthesia overdose using Zoletil 50^®^ (10 mg/kg BW) at the age of 6 weeks. Blood samples were collected from the abdominal aorta and centrifuged at 900× *g* for 10 min. The obtained serum was stored at −80 °C. We collected the colon, left kidney, heart, hypothalamus, prefrontal cortex, aortic arch, thoracic aorta, abdominal aorta, and approximately 1 cm × 1 cm of the left lateral lobe of the liver; these organs were then immediately frozen at −80 °C.

### 2.5. Total Tissue RNA Extraction, Reverse Transcription, and Real-Time Quantitative PCR

The Animal Total RNA Isolation Kit (Chengdu Foregene Biotech Co., Ltd., Chengdu, China) was used for total RNA extraction from the liver, left kidney cortex, left ventricle, hypothalamus, aortic arch, thoracic aorta, and abdominal aorta in accordance with the manufacturer’s instructions. Complementary DNA (cDNA) was then synthesized using the iScript cDNA Synthesis Kit (Bio-Rad Laboratories, Inc., Hercules, CA, USA). Real-time quantitative PCR (qPCR) was performed using SsoAdvanced Universal SYBR Green Supermix (Bio-Rad Laboratories, Inc.) and a CFX96 Touch Real-Time PCR System (Bio-Rad Laboratories, Inc.). We analyzed the expression levels of several components of the RAS, including angiotensin (*AGT*), renin (*Ren*), angiotensin-converting enzyme (*ACE*), angiotensin-converting enzyme II (*ACE2*), angiotensin II type I receptor (*AT*_1_*R*), and *Mas1* (receptor of angiotensin-(1-7)). *GAPDH* was selected as the internal control. Finally, the fold-changes in the experimental groups, relative to the control group, were calculated using the 2^−ΔΔCt^ method. Table S3 shows the reverse-transcription PCR protocol. Table S4 shows the qPCR protocol. Table S5 shows the primer sequences for qPCR.

### 2.6. Detection of Cytokines in the Colon and Prefrontal Cortex

IL-1β, IL-6, and TNF-α concentrations in the colon and prefrontal cortex were measured using ELISA kits from Elabscience Biotechnology Co., Ltd. (Wuhan, China). Briefly, 5 mg of colon tissue and 40 mg of prefrontal cortex were weighed, and 45 μL and 760 μL of Gibco PBS buffer (pH 7.4) were added to the organ samples, respectively. After homogenization at 5000× *g* for 10 min, the supernatants of the prefrontal cortex and colon were used for cytokine detection in accordance with the manufacturer’s instructions.

### 2.7. Fecal SCFA Analysis

For fecal samples collected at 3 weeks of age, 6 mixed samples of 100 mg in each group were prepared. For fecal samples collected at 6 weeks of age, 100 mg sample of each rat was prepared. Fecal SCFA analysis, including the determination of acetic acid, propionic acid, butyric acid, valeric acid, and isovaleric acid, was then conducted by Suzhou PANOMIX Biomedical Tech Co., Ltd. (Suzhou, China).

Samples were homogenized for 1 min with 500 μL of water and 100 mg of glass beads and then centrifuged at 13,800× *g* for 10 min at 4 °C. Next, 200 μL of supernatant was extracted with 100 μL of 15% phosphoric acid and 20 μL of 375 μg/mL 4-methylvaleric acid solution as IS and 280 μL of ether. Subsequently, the samples were centrifuged at 13,800× *g* for 10 min at 4 °C after vortexing for 1 min, and the supernatant was transferred into a vial prior to GC-MS analysis.

GC analysis was performed using a TRACE 1300 gas chromatograph (Thermo Fisher Scientific, Waltham, MA, USA). The GC was fitted with a capillary column Agilent HP-INNOWAX (30 m × 0.25 mm ID × 0.25 μm), and helium was used as the carrier gas at the flow rate of 1 mL/min. The sample was injected in the split mode at 10:1 with an injection volume of 1 μL and at an injector temperature of 250 °C. The temperatures of the ion source and interface were 300 °C and 250 °C, respectively. The column temperature was programmed to increase from an initial temperature of 90 °C, followed by an increase to 120 °C at 10 °C/min, then to 150 °C at 5 °C/min, and finally to 250 °C at 25 °C/min; this temperature was maintained for 2 min.

The mass spectrometric detection of metabolites was performed on ISQ 7000 (Thermo Fisher Scientific, Waltham, MA, USA) under the electron impact ionization mode. The single ion monitoring (SIM) mode was used with the electron energy of 70 eV.

### 2.8. Analysis of Serum TMAO and ITS precursors

Betaine, creatinine, carnitine, choline, trimethylamine (TMA), and TMAO levels in serum were quantitated by Applied Protein Technology (Zhejiang) Co., Ltd. (Yiwu, China) A serum sample (50 μL) was placed in a 2 mL centrifuge tube after thawing on ice, and 10 μL of internal standard solution and 0.45 mL of methanol were then added; the solutions were then mixed by vortex. After centrifugation at 14,000× *g*, the obtained supernatant was then subjected to HPLC–MS/MS analysis.

Separation was performed using a UPLC system (Agilent 1290 Infinity UHPLC, Waters, MA, USA) on a HILIC column (BEH HILIC 2.5 µm, 2.1 mm × 100 mm column, Waters) via gradient elution. Eluent A was acetonitrile, and Eluent B was water with 10 mM of ammonium formate buffer (pH 3.5). The gradient elution program was as follows: 0 min = 90% B, 1.5 min = 90% B, 4.5 min = 87% B, 7 min = 85% B, 7.5 min = 50% B, 10 min = 50% B, 10.5 min = 90% B, and 14 min = 90% B. Before injecting the next sample, the column was equilibrated with the initial mobile phase for 5 min. The flow rate was constant at 0.4 mL/min, and the column temperature was set at 25 °C.

Mass spectrometry was performed using the 5500 QTRAP system (AB SCIEX) in the positive switch mode. The ESI source conditions were as follows: source temperature: 550 °C; ion source gas1 (Gas1): 55; ion source gas2 (Gas2): 55; curtain gas (CUR): 40; and ion spray voltage floating (ISVF): +4500 V. The MRM method was used for mass spectrometry quantitative data acquisition.

### 2.9. Statistical Analysis

Data of male and female rats were analyzed using GraphPad Prism 8.0. Data are presented as mean ± SD (standard deviation). One-way ANOVA was performed for the data following Gaussian distribution and homogeneity of variance, and a post hoc test was then conducted using Tukey’s test. Otherwise, the Kruskal–Wallis test and the post hoc Dunn’s multiple comparisons test were used. Spearman’s rank correlation analysis was then conducted, and heatmaps of Spearman’s rank correlation matrices of male and female rats were constructed. A *p*-value of <0.05 was considered statistically significant.

## 3. Results

### 3.1. Effects of Early Life CEF and HFD Treatment on BW Increment

**Figure 1:**
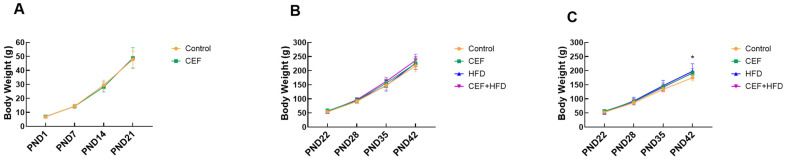
Body weight of (**A**) 0–3 weeks (Control *n* = 31, CEF *n* = 32), (**B**) 3–6 weeks of male rat (Control *n* = 6, CEF *n* = 7, HFD *n* = 8, CEF + HFD *n* = 7), and (**C**) 3–6 weeks of female rats (Control *n* = 9, CEF *n* = 9, HFD *n* = 8, CEF + HFD *n* = 9). CEF, ceftriaxone group. HFD, high-fat diet group. CEF + HFD, ceftriaxone followed with high-fat diet group. PND: postnatal day. * *p* < 0.05 between Control vs. CEF.

The BW of newborn rats increased steadily before and after weaning (Figure 1). Since the sex of new born pups cannot be determined at all after birth immediately by visual observation, the data of BW of both male and female rats were recorded together before 3 weeks (Figure 1A). No significant difference in BW was observed between the CEF and control groups at 3 weeks of age (Figure 1A). Similar results were noted for male rats at 6 weeks of age (Figure 1B). For female rats at 6 weeks of age (Figure 1C), the BW of the control group was slightly lower than those of the other groups, and a significant difference in BW was observed between the CEF and control groups (*p* < 0.05).

### 3.2. Effects of Early-Life CEF and HFD Treatment on SBP and DBP

**Figure 2:**
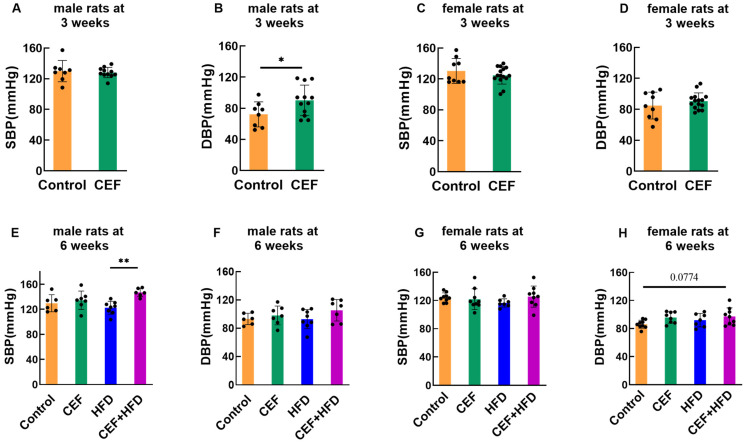
Systolic blood pressure (SBP) and diastolic blood pressure (DBP) of the rats. (**A**,**B**) male rats at the age of 3 weeks (Control *n* = 8, CEF *n* = 12), (**C**,**D**) female rats at the age of 3 weeks (Control *n* = 9, CEF *n* = 15), (**E**,**F**) male rats at the age of 6 weeks (Control *n* = 6, CEF *n* = 7, HFD *n* = 8, CEF + HFD *n* = 7), (**G**,**H**) female rats at the age of 6 weeks (Control *n* = 9, CEF *n* = 9, HFD *n* = 8, CEF + HFD *n* = 9). CEF ceftriaxone group, HFD high-fat diet group, CEF + HFD ceftriaxone and high-fat diet group. * *p* < 0.05, ** *p* < 0.01.

By 3 weeks of age, ceftriaxone had no significant effect on the SBP of male and female rats (Figure 2A,C); however, ceftriaxone increased DBP to some extent, and a significant change was observed in male rats (Figure 2B,D).

At 6 weeks of age, ceftriaxone alone slightly increased SBP and DBP in both male and female rats; however, no significant change was detected. HFD alone rarely affected the SBP and DBP values of rats. However, compared to the other groups, male rats in the CEF + HFD group showed increased SBP and DBP values, and a significant increase was observed compared to the HFD group. CEF + HFD treatment also affected the DBP of female rats (*p* < 0.1) (Figure 2E–H).

### 3.3. Modulation of the RAS in Different Tissues after Eraly Life CEF and HFD Treatment

In male rats (Table 1), the expression levels of some genes of the RAS were affected in the left kidney, heart, hypothalamus, thoracic aorta, and abdominal aorta. Ceftriaxone treatment in early life significantly decreased the expression of *Ren* in the left kidney of the CEF group (*p* < 0.05) when compared to that in the control group. HFD feeding significantly increased the expression of *AT*_1_*R* in the hypothalamus of the HFD group (*p* < 0.05) compared to that in the control group. Overall, ceftriaxone or HFD alone had limited effects on the functioning of the RAS in different tissues. However, compared to the control group, the combination of both ceftriaxone and HFD had wide effects on the RAS in different tissues, e.g., decreased expression of *Ren* in the left kidney, increased expression of *ACE* and *AT*_1_*R* in the hypothalamus, and increased expression of *Mas1* in the abdominal aorta (*p* < 0.05).

Table 2 shows the relative expression levels of RAS genes in female rats. No significant difference was observed between the CEF and control groups. The HFD group showed a significant upregulation of *Ren* and *ACE* expression in the kidney compared to that in the control group (*p* < 0.05). Ceftriaxone treatment followed by HFD feeding decreased *ACE* expression in the kidney and heart (*p* < 0.05).

### 3.4. Changes in the Concentrations of Cytokines in the Colon and Prefrontal Cortex

**Figure 3:**
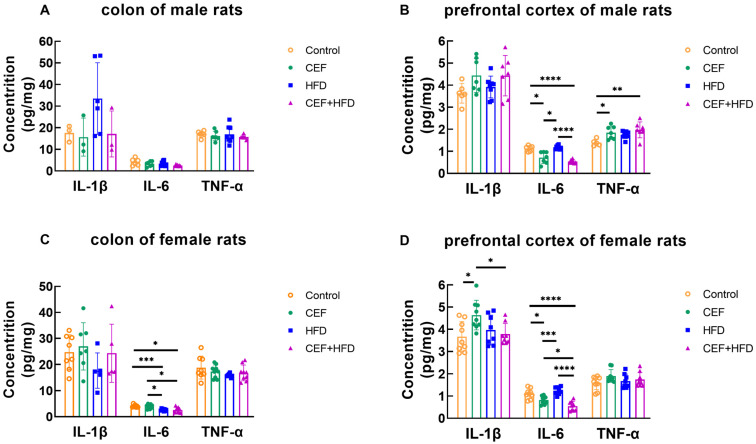
Concentrations of cytokines in colon and prefrontal cortex of (**A**,**B**) male rats at 6 weeks of age (Control *n* = 6, CEF *n* = 7, HFD *n* = 8, CEF + HFD *n* = 7) and (**C**,**D**) female rats at 6 weeks of age (Control *n* = 9, CEF *n* = 9, HFD *n* = 8, CEF + HFD *n* = 9). CEF, ceftriaxone group. HFD, high-fat diet group. CEF + HFD, ceftriaxone and high-fat diet group. * *p* < 0.05, ** *p* < 0.01, *** *p* < 0.001, **** *p* < 0.0001.

Figure 3 shows the concentrations of IL-1β, IL-6, and TNF-α detected in the colon and prefrontal cortex. No significant change in cytokine concentration was observed in the colon of male rats (Figure 3A). In the prefrontal cortex of male rats, compared to the control and HFD group, the CEF and CEF + HFD groups showed decreased levels of IL-6 and increased levels of TNF-α (Figure 3B). In the colon of female rats, IL-6 levels were decreased in rats fed with HFD compared to those in rats fed with a regular diet (Figure 3C). Furthermore, in the prefrontal cortex of female rats, the trend observed in the IL-6 levels was similar to that observed in male rats, while a higher level of IL-1β was found in the CEF group (Figure 3D).

### 3.5. Alterations in Gut Microbiota Diversity after Early-Life CEF and HFD Treatment

**Figure 4:**
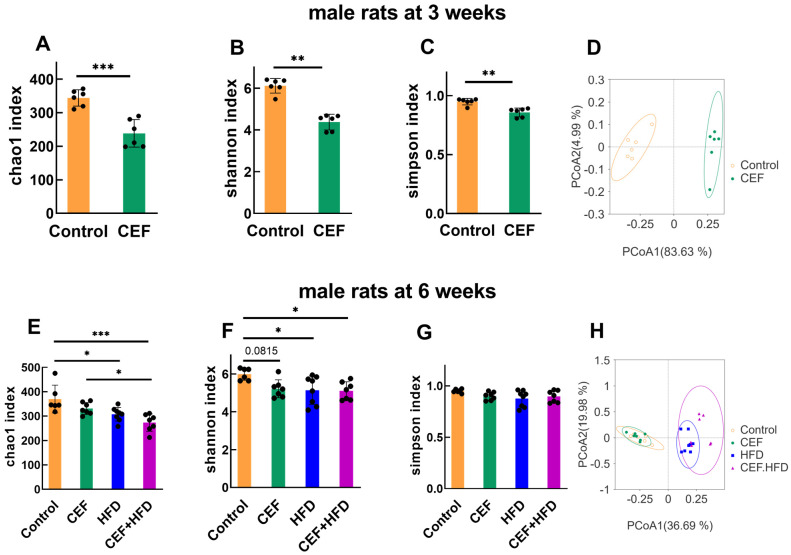
α diversity and principal coordinate analysis (PCoA) of fecal microbiota at the age of 3 and 6 weeks in male rats. (**A**–**C**) α diversity at 3 weeks of age (Control *n* = 6, CEF *n* = 6). (**D**) PCoA of fecal microbiota at 3 weeks of age. (**E**–**G**) α diversity at 6 weeks of age (Control *n* = 6, CEF *n* = 7, HFD *n* = 8, CEF + HFD *n* = 7). (**H**) PCoA of fecal microbiota at 6 weeks of age. CEF, ceftriaxone group. HFD, high-fat diet group. CEF + HFD, ceftriaxone and high-fat diet group. * *p* < 0.05, ** *p* < 0.01, *** *p* < 0.001.

Three major parameters, namely the Chao1 index, Shannon index, and Simpson index, were used to evaluate the richness, evenness, and diversity of gut microbial species, respectively. In male rats at 3 weeks of age, these three parameters were remarkably decreased after ceftriaxone treatment (*p* < 0.001 for Chao1 index, and *p* < 0.01 for Shannon and Simpson indices), thus suggesting a reduction in gut microbial species richness, evenness, and diversity (Figure 4A–C). The PCoA map showed a drastic separation of gut microbial species between the CEF and control groups (Figure 4D).

In male rats at 6 weeks of age, the Chao1 and Shannon indices were significantly decreased by HFD alone or in combination with ceftriaxone compared to those in the control group (Figure 4E,F). However, the CEF group showed no significant decrease in the Chao1 and Shannon indices compared to those in the control group (Figure 4E,F). The groups showed slight effects on the Simpson index (Figure 4G). The PCoA map showed two separate clusters between rats fed with a regular diet and HFD (Figure 4H); this difference might be because the HFD after ceftriaxone treatment hindered the recovery of gut microbiota from dysbiosis due to ceftriaxone exposure.

**Figure 5:**
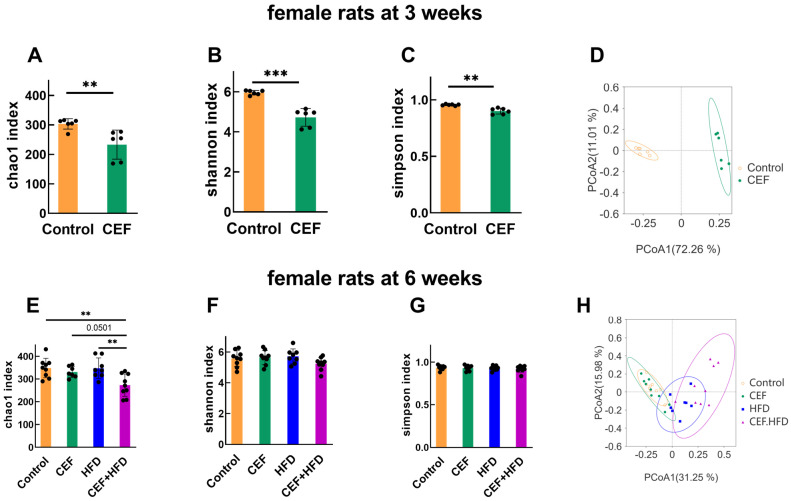
α diversity and principle coordinate analysis (PCoA) of fecal microbiota at the age of 3 and 6 weeks of female rats. (**A**–**C**) α diversity at 3 weeks of age (Control *n* = 6, CEF *n* = 6). (**D**) PCoA of fecal microbiota at 3 weeks of age. (**E**–**G**) α diversity at 6 weeks of age (Control *n* = 9, CEF *n* = 9, HFD *n* = 8, CEF + HFD *n* = 9). (**H**) PCoA of fecal microbiota at 6 weeks of age. CEF, ceftriaxone group. HFD, high-fat diet group. CEF + HFD, ceftriaxone and high-fat diet group. ** *p* < 0.01, *** *p* < 0.001.

In female rats, at the age of weaning, the three parameters evaluating gut microbial species were decreased remarkably, and the PCoA map showed a significant cluster separation between the CEF and control groups (Figure 5A–D); these results were almost identical to those observed in male rats. However, at the age of 6 weeks, except for an evident decrease in Chao1 index in the CEF + HFD group, no other significant change was observed (Figure 5E–G). Interestingly, the PCoA map showed some overlapping areas among all the four groups (Figure 5H). These findings suggest that female rats suffer less from HFD-induced adversity and recover more easily in terms of gut microbial species richness, evenness, and diversity than male rats.

### 3.6. Alterations in the Relative Abundance of GUT Microbiota after Early-Life CEF and HFD Treatment

**Figure 6:**
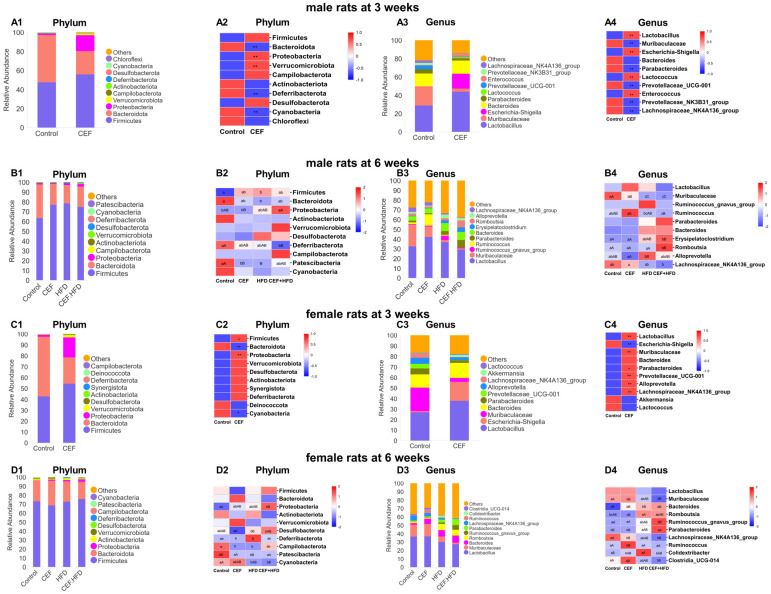
Relative abundance and heat map of gut microbiota for the top 10 categories of gut microbiota in (**A1**–**A4**) male rats at 3 weeks of age (Control *n* = 6, CEF *n* = 6), (**B1**–**B4**) male rats at 6 weeks of age (Control *n* = 6, CEF *n* = 7, HFD *n* = 8, CEF + HFD *n* = 7), (**C1**–**C4**) female rats at 3 weeks of age (Control *n* = 6, CEF *n* = 6), and (**D1**–**D4**) female rats at 6 weeks of age (Control *n* = 9, CEF *n* = 9, HFD *n* = 8, CEF + HFD *n* = 9). CEF, ceftriaxone group. HFD, high-fat diet group. CEF + HFD, ceftriaxone and high-fat diet group. * *p* < 0.05 and ** *p* < 0.01 for data of 3 weeks. No common small letter means *p* < 0.05 and no common capital letter means *p* < 0.01 for data of 6 weeks.

Figure 6 shows the histograms and heatmaps of the relative abundance of gut microbiota in different time periods. In male rats at 3 weeks of age, an increase was observed in the phyla *Firmicutes* and *Proteobacteria*, while a decrease was noted in the phylum *Bacteroidota* in the CEF group, which resulted in an increased *Firmicutes* to *Bacteroidetes* (F/B) ratio (Figure 6(A1,A2)). At the genus level, *Lactobacillus* and *Escherichia-Shigella* showed an increased abundance, accompanied with a decrease in *Muribaculaceae* (Figure 6(A3,A4)). At 6 weeks of age, the F/B ratios were increased in all the three experimental groups (Figure 6(B1,B2)). At the genus level, all the three experimental groups showed a decrease in the abundance of *Muribaculaceae*, while *Romboutsia* increased significantly in the CEF + HFD group compared to that in the control group (Figure 6(B3,B4)).

The F/B ratio of female rats also increased in the CEF group at 3 weeks of age (Figure 6(C1,C2)). At the genus level, a higher abundance of *Lactobacillus* and *Bacteroides*, a much higher abundance of *Escherichia-Shigella*, and a lower abundance of *Muribaculaceae* were observed (Figure 6(C3,C4)). However, at 6 weeks of age, there were less changes in *Firmicutes* and *Bacteroidota*, resulting in slight fluctuations in the F/B ratio in the three experimental groups compared to that in the control group (Figure 6(D1,D2)). At the genus level, *Muribaculaceae* decreased after HFD intervention, while the number of *Bacteroides* increased in all the three experiment groups compared to that in the control group (Figure 6(D3,D4)).

### 3.7. Changes in the Level of Fecal SCFAs after Early-Life CEF and HFD Treatment

**Figure 7:**
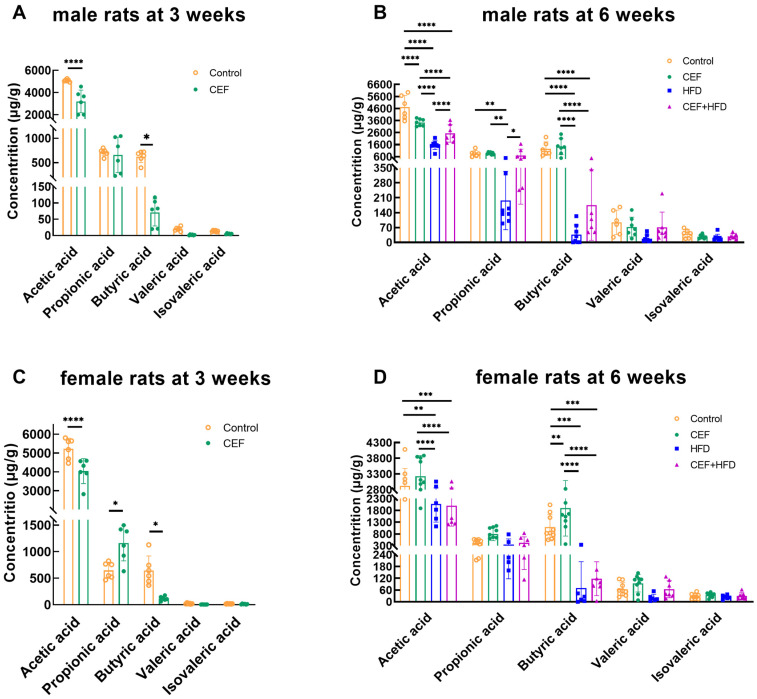
Concentrations of SCFAs of feces in (**A**) male rats at 3 weeks of age (Control *n* = 6, CEF *n* = 6), (**B**) male rats at 6 weeks of age (Control *n* = 6, CEF *n* = 7, HFD *n* = 8, CEF + HFD *n* = 7), (**C**) female rats at 3 weeks of age (Control *n* = 6, CEF *n* = 6), and (**D**) female rats at 6 weeks of age (Control *n* = 9, CEF *n* = 9, HFD *n* = 8, CEF + HFD *n* = 9). CEF, ceftriaxone group. HFD, high-fat diet group. CEF + HFD, ceftriaxone and high-fat diet group. * *p* < 0.05, ** *p* < 0.01, *** *p* < 0.001, **** *p* < 0.0001.

Figure 7 shows the concentrations of fecal SCFAs, including acetic acid, propionic acid, butyric acid, valeric acid, and isovaleric acid. In male rats at 3 weeks of age, ceftriaxone treatment decreased the concentration of acetic acid (*p* < 0.0001) and butyric acid (*p* < 0.05) (Figure 7A). At 6 weeks of age, the concentration of only acetic acid decreased in the CEF group compared to that in the control group (*p* < 0.05). The concentrations of acetic, propionic, and butyric acids decreased to varying degrees following HFD feeding, regardless of whether it was combined or not combined with ceftriaxone treatment. Interestingly, the HFD group showed the lowest concentration of SCFAs, which was significantly lower than that of the CEF + HFD group (Figure 7B).

In female rats at 3 weeks of age, the concentrations of acetic acid and butyric acid were decreased (*p* < 0.0001 and *p* < 0.05, respectively), and the concentration of propionic acid was increased (*p* < 0.05) in the CEF group compared to those in the control group (Figure 7C). At 6 weeks of age, the concentrations of acetic acid and butyric acid showed significant changes (Figure 7D). More specifically, ceftriaxone treatment increased the concentration of butyric acid (*p* < 0.01), while the concentrations of acetic acid and butyric acid decreased after HFD feeding, regardless of whether it was combined or not combined with ceftriaxone treatment. These findings were consistent with the results of the PCoA maps shown in Figure 4 and Figure 5. However, no significant change was found between the HFD and CEF + HFD groups, which differed from the results noted in male rats.

### 3.8. Changes in the Levels of Serum TMAO and Its Precursors after Early-Life CEF and HFD Treatment

**Figure 8:**
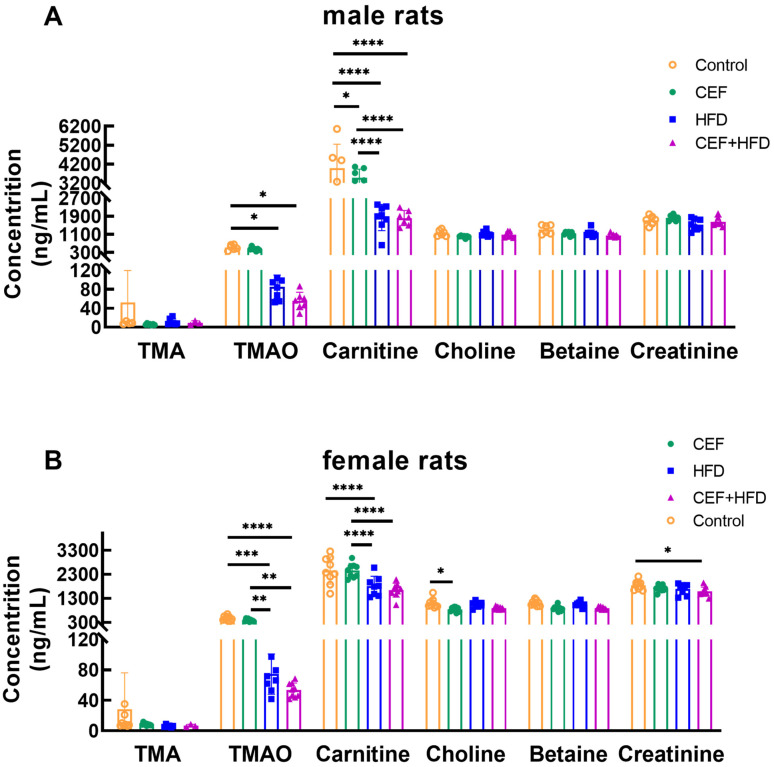
Concentrations of serum TMAO and its relative components in (**A**) male rats (Control *n* = 6, CEF *n* = 7, HFD *n* = 8, CEF + HFD *n* = 7) and (**B**) female rats (Control *n* = 9, CEF *n* = 9, HFD *n* = 8, CEF + HFD *n* = 9). CEF, ceftriaxone group. HFD, high-fat diet group. CEF + HFD, ceftriaxone and high-fat diet group. * *p* < 0.05, ** *p* < 0.01, *** *p* < 0.001 **** *p* < 0.0001.

Figure 8 shows the serum concentrations of TMAO and its precursors. In both male and female rats, serum TMA concentrations were decreased in all the three experimental groups compared to those in the control group, although the difference was not significant; this might be because the perturbed gut microbiota could hardly transform some compounds from the diet, such as choline and betaine, into TMA. The serum TMAO concentrations were significantly decreased after HFD feeding, regardless of whether it was combined or not combined with ceftriaxone treatment. This might be because of the low content of ingredients producing TMA and TMAO in HFD fodder.

### 3.9. Spearman’s Rank Correlation Analysis between SBP/DBP and Biochemical Indicators

**Figure 9:**
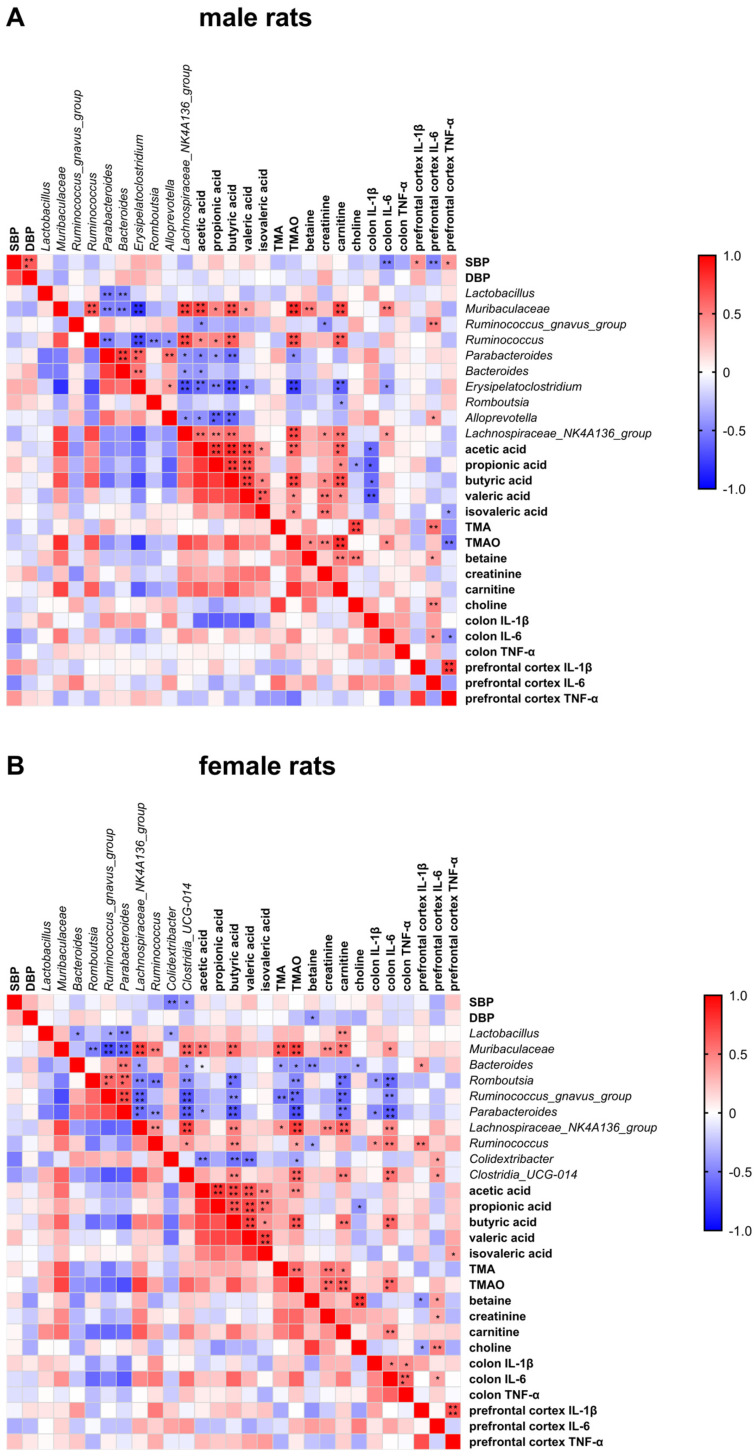
Spearman rank correlative matrix between SBP/DBP and biochemical indicators including gut microbiota, SCFAs, TMAO and precursors, and cytokines in (**A**) male rats and (**B**) female rats. SBP, systolic blood pressure. DBP, diastolic blood pressure. TMA, trimethylamine, TMAO, trimethylamine N-oxide. * *p* < 0.05, ** *p* < 0.01, *** *p* < 0.001, **** *p* < 0.0001.

Figure 9 shows Spearman’s rank correlation matrices between SBP/DBP and biochemical indicators, including the relative abundance of gut microbiota, SCFAs, TMAO and its precursors, and cytokines, in male and female rats.

In male rats (Figure 9A), SBP was negatively associated with IL-6 levels in the colon and prefrontal cortex, but it was positively associated with IL-1β and TNF-α levels in the prefrontal cortex. Several gut microbial taxa were associated with three or four types of fecal SCFAs, e.g., *Muribaculaceae*, *Ruminococcus*, *Parabacteroides*, *Erysipelatoclostridium*, *Alloprevotella*, and *Lachnospiraceae_NK4A136_group*, among which *Muribaculaceae* and *Ruminococcus* were positively associated, while others were negatively associated with the levels of SCFAs. *Muribaculaceae* was positively associated, but *Parabacteroides* was negatively associated with serum TMAO concentration. Finally, a negative correlation between the colon IL-6 level and *Erysipelatoclostridium*, a positive correlation between the colon IL-6 level and *Muribaculaceae* and *Lachnospiraceae_NK4A136_group*, and a correlation between the prefrontal cortex IL-6 level and *Ruminococcus_gnavus_group* and *Alloprevotella* were observed.

Female rats (Figure 9B) showed inverse correlations between SBP and *Colidextribacter* and *Clostridia_UCG-014*. *Muribaculaceae* and *Lachnospiraceae_NK4A136_group* were positively associated with the levels of SCFAs, particularly butyric acid, and TMAO with its precursors. However, inverse associations were found for *Romboutsia*, *Ruminococcus_gnavus_group*, and *Parabacteroides*. In particular, *Colidextribacter* was negatively associated with the levels of acetic, butyric, and valeric acids and TMAO, while *Clostridia_UCG-014* was positively associated with the levels of butyric acid, TMAO, and colonic IL-6. Both these taxa were positively associated with the IL-6 level in the prefrontal cortex.

## 4. Discussion

Our present study revealed an association between gut microbiota and pediatric blood pressure regulation in rats with a sex-specific difference. Overall, the perturbed gut microbiota in early life after HFD feeding might elevate SBP, particularly in males. Moreover, the gut microbiota of male rats seemed to be affected more by antibiotic exposure and HFD than that of female rats.

It is known that sex-specific differences exist in the progression and epidemiology of hypertension. Females have a lower risk of developing cardiovascular diseases than males; however, this advantage diminishes with the increase in age [16,17]. These sex-specific differences are reflected in gut microbiome composition, with an acute increase in the F/B ratio in male hypertensive patients compared to that in female hypertensive patients [18], a more intense immune response in males than in females [19], and a lower expression of *AT*_1_*R* in females than in males [20]. In the present study, sex-dependent differences were observed in pediatric blood pressure regulation, reflecting mainly more intense gut dysbiosis and more sensitive responses in the regulation of RAS and inflammation in males than in females.

### 4.1. Sexual Differences in the Changes in Pediatric Blood Pressure

In our present research, although all the rats did not meet the diagnostic criteria of hypertension (usually SBP > 150 mmHg in rats [21,22]), ceftriaxone treatment increased the DBP of male rats at 3 weeks of age, while only ceftriaxone followed by HFD feeding increased the SBP of male rats at 6 weeks of age. These results demonstrated that sexual differences exist in pediatric blood pressure regulation, and males are more susceptible to pediatric hypertension than females. In this study, HFD did not lead to obesity by 6 weeks of age, while rats with normal BW showed increased blood pressure; these findings indicate that a higher blood pressure might originate prior to obesity in the early stage of hypertension, considering that obesity is a risk factor and a major comorbidity of hypertension [23].

### 4.2. RAS Shows More Activation in Males than in Females during Pediatric Blood Pressure Regulation

The RAS participates in blood pressure regulation, and its components are expressed in many tissues. To date, five RAS pathways have been identified, with two core functional pathways [24]. Among the core functional pathways, the first one is the classic ACE-Ang II-AT_1_R axis, whose activation contributes to elevated blood pressure and vascular dysfunction [25], and the second one is the nonclassical ACE II-Ang-(1-7))-MasR axis, which helps lower blood pressure and inhibit vasoconstriction [26]. The hypothalamus is considered to be the blood-pressure-regulating center [27,28]. In the present study, the increased expression of *ACE* and *AT*_1_*R* in the hypothalamus of male rats might be partially responsible for the elevated blood pressure in the CEF + HFD group.

In male rats, changes in the RAS components were reflected in many tissues, including the kidney, heart, hypothalamus, thoracic aorta, and abdominal aorta. However, in female rats, changes in the RAS components were observed only in the kidney, heart, and hypothalamus. This finding suggests that the blood pressure of male rats is more sensitive to ceftriaxone and HFD exposure, because the RAS in the aortas of male rats showed some responses to the interventions, whereas no significant change was found in the RAS in the aortas of female rats.

### 4.3. Sex-Specific Changes in Proinflammatory Cytokine Levels Are Related to Pediatric Blood Pressure Regulation

Elevated levels of cytokines are associated with higher blood pressure and end-organ damage in hypertensive patients. IL-1β is produced by macrophages and monocytes, and it can promote the release of IL-6 [29]. The pre-activation of peripheral blood monocytes and increased levels of IL-1β and TNF were observed in hypertensive patients [30]. IL-1β and TNF-α may also promote endothelin-mediated vasoconstriction [31]. IL-6 can function as a proinflammatory or anti-inflammatory cytokine depending on the different pathways. The proinflammatory function of IL-6 is mainly driven by IL-6 trans-signaling, while the anti-inflammatory function relies on classic IL-6 signaling [32]. In our research, IL-6 levels in both the colon and prefrontal cortex of rats treated with ceftriaxone and HFD were significantly decreased compared to those in the control group; this finding contradicted the changes observed in SBP. This might be because these rats had not yet reached the stage of hypertension; thus, the anti-inflammatory function of IL-6 is activated more to maintain homeostasis when the rats are young. It could be reasonably hypothesized that IL-6 increases and becomes proinflammatory with the continuous exposure to ceftriaxone and HFD and the enhancement of IL-6 trans-signaling [33,34]. Furthermore, the IL-6 level in the colon and prefrontal cortex was negatively associated, whereas the IL-1β and TNF-α levels in the prefrontal cortex were positively associated with SBP in male rats in childhood. However, no correlation was observed between the levels of proinflammatory cytokines and SBP/DBP in female rats at 6 weeks of age. This finding indicated that changes in the levels of cytokines are also sex-specific, with males being more sensitive to these changes than females during childhood.

### 4.4. Early-Life Gut Microbiota of Males Is More Susceptible than That of Females

Exposure to antibiotics in early life is associated with obesity, allergic diseases, etc. [35,36]. However, the association between antibiotics and blood pressure is not consistent. For example, one study showed that maternal exposure to minocycline induces increased the SBP and F/B ratio of the gut microbiota in offspring [7], whereas another study revealed that minocycline attenuated hypertension and restored the dysbiosis of gut microbiota in rats with Ang II infusion hypertension [37]. Doxycycline also led to a recovery from gut dysbiosis and alleviated hypertension in deoxycorticosterone acetate (DOCA)-salt rats [38]. In other words, different regulating mechanisms might exist between developing and developed hypertension via the unbalancing or rebalancing of the gut microbiota using antibiotics. In addition, antibiotics themselves are rarely used to treat hypertension [39]. Therefore, we chose ceftriaxone sodium, an antibiotic that is not absorbed in the intestine, to cause gut dysbiosis.

Our present research revealed an association between gut microbiota and blood pressure of rats in childhood. At 3 weeks of age, both male and female rats showed decreased α diversity and an increased F/B ratio after ceftriaxone treatment. However, in male rats at 6 weeks of age, Chao1 and Shannon indices were still lower and the F/B ratio was still higher after a regular diet or HFD feeding when compared to those in the control group; meanwhile, in female rats, these indices showed recovery to various degrees, regardless of the diet. This finding indicates that antibiotic exposure could have longer effects on male rats than on female rats, and that the gut microbiota of female rats is preserved to a greater extent than that of male rats. These results are consistent with the findings of Mushtaq et al. [18], who found an acute increase in the F/B ratio in male hypertensive patients compared to that in female patients. Some studies have suggested that the low richness and diversity of the gut microbiota are associated with hypertension [37,40]. However, Hsu et al. found an elevated α-diversity in hypertensive rats induced by minocycline; this result indicated that α-diversity might not be a crucial factor for the development of hypertension [7]. Thus, there might exist special gut microbiota related to pediatric blood pressure regulation.

### 4.5. Gut Microbiota Related to Pediatric Blood Pressure Regulation Were Found Only in Female Rats

Both male and female rats showed similar changes in several gut microbial taxa. In general, the abundance of *Muribaculaceae* decreased in both male and female rats at 6 weeks of age, while the abundance of *Parabacteroides*, *Bacteroides*, and *Romboutsia* increased. *Muribaculaceae* is one of the predominant microbiotas in the phylum *Bacteroidetes* [41]. An increase in dietary fat could lead to a decrease in *Muribaculaceae* [42], whereas an increased abundance of *Muribaculaceae* was observed in studies on the mitigation of obesity [43,44]. *Muribaculaceae* might help to combat obesity. The relationships between *Parabacteroides* and health conditions such as obesity, metabolic syndrome, and inflammatory bowel disease were reviewed by Cui et al. [45]. *Parabacteroides merdae*, an opportunistic pathogen, was found to be frequently distributed in the hypertensive gut microbiome [46]. Even though most of these gut microbial taxa with similar alteration trends were found to have limited association with pediatric blood pressure regulation, their potential to mediate obesity might also cause hypertension in later life.

A sex-specific difference was noted in the negative relationship between SBP and *Colidextribacter* and *Clostridia_UCG-014* in female rats at 6 weeks of age, even though these rats did not have hypertension. *Colidextribacter*, an HFD-dependent species, could be promoted by a high-fat and high-cholesterol diet [47,48]. A higher abundance of *Clostridia_UCG-014* induced by a HFD was also observed [49]. However, it was found to have a negative association with serum lipid levels [42]. The changes in the abundance of *Colidextribacter* and *Clostridia_UCG-014* prior to the development of hypertension could serve as a biomarker of hypertension. Overall, few gut microbial taxa were found to be associated with blood pressure changes. One of the main reasons for this might be that the body during childhood still has self-regulating capacity while developing hypertension. Another reason might be that the gut microbiota itself has limited effects on blood pressure directly, while its metabolites might exert greater effects through the gut–brain axis or immune response activation.

### 4.6. Sex-Specific Differences Are Reflected in the Levels of SCFAs Rather than in TMAO in Pediatric Blood Pressure Regulation

SCFAs, mainly produced by high-fiber foods, are thought to play a role in maintaining blood pressure balance. It is hypothesized that the loss of SCFA-producing bacteria, which causes a decrease in the levels of acetate, propionate, and butyrate, is one of the pathogenetic mechanisms of hypertension [50]. SCFAs, after absorption, participate in blood pressure regulation through binding with G protein-coupled receptors such as Gpr41, Gpr43, Gpr109a, and Olfr78, which are widely distributed in the kidney, brain, cardiac tissue, and vasculature [51]. SCFAs can also regulate the function of immune cells and further affect blood pressure. In the present study, at 3 weeks of age, ceftriaxone treatment decreased the levels of acetic and butyric acids in both male and female rats, except for an increase in propionic acid level in female rats. However, the sex-specific differences were more apparent at 6 weeks of age. HFD feeding decreased the levels of acetic and butyric acids in both male and female rats, while propionic acid was decreased only in male rats. This finding indicated that HFD might affect the gut microbiota of female rats to a lesser extent. The significant differences in fecal SCFAs between the HFD group and other groups in male rats suggest the potential longer effects of antibiotics on male rats than on female rats. However, the relationship between SBP/DBP and SCFAs in childhood remains unclear.

TMA, another gut microbial metabolite, is produced from betaine, carnitine, and choline, which are rich in saltwater fish, eggs, red meat, and dairy products [52]. TMA is oxidized to TMAO by FMOs in the liver [12]. TMAO plays a role in inflammation, obesity, and cardiovascular diseases [53,54,55], and a positive correlation was observed between TMAO and SBP, and between TMAO and hypertension in humans [56,57]. However, in the present study, neither sex-specific differences in TMAO and its precursors, nor the relationship between SBP/DBP and TMAO with its precursors, was found. The sex-specific differences in TMAO and its precursors might occur during sexual maturity and hormone secretion [58]. Overall, further studies are required to determine how SCFAs and TMAO participate in pediatric blood pressure regulation.

## 5. Conclusions

This research focused on how gut dysbiosis induced by ceftriaxone and HFD during early life could affect blood pressure in childhood in different ways and in different genders. Gut dysbiosis in early life could increase SBP in juvenile rats, and this was more evident in male rats. Male rats were more sensitive and susceptible to RAS dysfunction and chronic inflammation. However, it remains unclear how the gut microbial metabolites play a role in pediatric blood pressure regulation. Fecal microbiota transplantation from antibiotics or HFD subjects to normal subjects or germ-free animals may be an effective method by which to discover the cause of pediatric blood pressure changes. Moreover, further investigations are required to determine how SCFAs regulate blood pressure by mediating G protein-coupled receptors or immune responses, and how sex-specific changes in TMAO with its precursors, as well as end-organ injuries such as intestinal epithelial injury, in early life affect pediatric blood pressure regulation or the early stage of hypertension.

## Figures and Tables

**Table 1:** The relative expressions of genes in RAS in different tissues of male rats at 6 weeks of age.

Group		Control	CEF	HFD	CEF + HFD
liver	*AGT*	1.77 ± 1.07a	1.91 ± 0.97a	1.13 ± 0.80a	1.49 ± 0.92a
cortex of left kidney	*Ren*	**3.94 ± 2.43a**	**0.63 ± 0.66b**	**2.21 ± 1.32ab**	**0.97 ± 1.10b**
	*ACE*	1.87 ± 1.02a	1.98 ± 1.71a	1.53 ± 0.74a	0.64 ± 0.62a
	*ACE2*	1.84 + 1.38a	1.27 ± 0.53a	1.91 ± 1.55a	1.44 ± 0.92a
	*AT*_1_ *R*	1.21 ± 0.35a	1.29 ± 0.24a	0.87 ± 0.22a	1.28 ± 0.40a
	*Mas1*	1.22 ± 0.38a	1.14 ± 0.24a	1.01 ± 0.26a	1.30 ± 0.24a
left ventricle	*ACE*	**2.41 ± 1.66ab**	**1.44 ± 0.21a**	**1.67 ± 0.93a**	**0.10 ± 0.04b**
	*ACE2*	1.62 ± 1.18a	0.79 ± 0.55a	1.30 ± 1.22a	1.03 ± 0.32a
	*AT*_1_ *R*	1.60 ± 0.64a	1.92 ± 1.04a	2.19 ± 0.77a	1.89 ± 0.63a
	*Mas1*	**1.23 ± 0.42ab**	**1.84 ± 0.61a**	**0.85 ± 0.25b**	**1.59 ± 0.51a**
hypothalamus	*ACE*	**1.09 ± 0.24b**	**1.91 ± 1.28ab**	**1.30 ± 0.51ab**	**2.58 ± 1.26a**
	*ACE2*	0.92 ± 0.53a	1.31 ± 0.57a	1.82 ± 1.39a	1.77 ± 1.02a
	*AT*_1_ *R*	**0.68 ± 0.32a**	**1.96 ± 1.62ab**	**1.90 ± 0.80b**	**1.61 ± 0.47b**
	*Mas1*	1.77 ± 1.19a	1.45 ± 0.80a	1.26 ± 0.61a	2.50 ± 1.51a
aortic arch	*ACE*	3.63 ± 1.81a	1.51 ± 1.16a	4.46 ± 4.04a	1.89 ± 2.13a
	*ACE2*	0.87 ± 0.45a	0.86 ± 0.49a	1.48 ± 1.11a	1.29 ± 0.79a
	*AT*_1_ *R*	0.83 ± 0.46a	0.62 ± 0.19a	1.30 ± 0.74a	0.67 ± 0.34a
	*Mas1*	1.04 ± 0.22a	0.76 ± 0.46a	0.98 ± 0.35a	1.09 ± 0.55a
thoracic aorta	*ACE*	0.42 ± 0.34a	ND	1.55 ± 1.30a	12.27 ± 12.25a
	*ACE2*	0.63 ± 0.55a	1.05 ± 0.76a	0.32 ± 0.19a	1.17 ± 0.66a
	*AT*_1_ *R*	0.92 ± 0.80a	2.32 ± 2.03a	0.81 ± 0.63a	2.62 ± 2.25a
	*Mas1*	**0.97 ± 0.80ab**	**2.13 ± 1.08a**	**0.57 ± 0.19b**	**1.71 ± 0.87ab**
abdominal aorta	*ACE*	3.25 ± 2.38a	1.14 ± 1.35a	1.42 ± 1.19a	0.52 ± 0.25a
	*ACE2*	0.30 ± 0.23a	ND	0.07 ± 0.04a	0.33 ± 0.17a
	*AT*_1_ *R*	**1.10 ± 0.81ab**	**1.69 ± 0.84a**	**0.65 ± 0.35b**	**1.32 ± 0.63ab**
	*Mas1*	**0.64 ± 0.31b**	**0.62 ± 0.18b**	**1.06 ± 0.43ab**	**1.33 ± 0.39a**

Control, *n* = 6. CEF, ceftriaxone group, *n* = 7. HFD, high-fat diet group, *n* = 8, CEF + HFD, ceftriaxone and high-fat diet group, *n* = 7. No common letter in each row means *p* < 0.05. ND, not detected. The changed genes were emphasized by bold face font.

**Table 2:** The relative expression of genes in RAS in different tissues of female rats at 6 weeks of age.

Group		Control	CEF	HFD	CEF + HFD
liver	*AGT*	1.02 ± 0.61a	0.85 ± 0.33a	1.03 ± 0.44a	2.62 ± 1.67a
cortex of left kidney	*Ren*	1.70 ± 1.48a	1.58 ± 1.72a	10.86 ± 11.05b	0.44 ± 0.52a
	*ACE*	**1.27 ± 0.96b**	**0.86 ± 0.54bc**	**4.10 ± 2.93a**	**0.41 ± 0.39c**
	*ACE2*	1.11 ± 0.48a	0.96 ± 0.49a	1.83 ± 1.3a	1.14 ± 0.79a
	*AT*_1_ *R*	1.13 ± 0.50a	1.02 ± 0.20a	1.08 ± 0.33a	0.89 ± 0.24a
	*Mas1*	1.08 ± 0.42a	1.10 ± 0.43a	1.13 ± 0.37a	1.17 ± 0.27a
left ventricle	*ACE*	**1.23 ± 0.75a**	**1.66 ± 1.62a**	**2.03 ± 1.21a**	**0.14 ± 0.11b**
	*ACE2*	0.80 ± 0.33a	1.03 ± 0.67a	1.14 ± 0.75a	0.84 ± 0.22a
	*AT*_1_ *R*	1.47 ± 1.13a	1.24 ± 0.78a	1.89 ± 1.01a	1.29 ± 0.54a
	*Mas1*	**1.10 ± 0.47ab**	**1.14 ± 0.57ab**	**0.71 ± 0.20a**	**1.35 ± 0.47b**
hypothalamus	*ACE*	**0.90 ± 0.25ab**	**0.92 ± 0.50ab**	**0.84 ± 0.20a**	**1.36 ± 0.41b**
	*ACE2*	0.97 ± 0.50a	1.42 ± 1.22a	0.82 ± 0.55a	1.10 ± 0.48a
	*AT*_1_ *R*	1.16 ± 0.64a	1.21 ± 0.79a	1.08 ± 0.42a	0.74 ± 0.29a
	*Mas1*	1.28 ± 0.85a	1.19 ± 0.70a	2.62 ± 1.16a	2.11 ± 1.49a
aortic arch	*ACE*	1.23 ± 0.91a	0.90 ± 0.72a	4.41 ± 4.61a	2.18 ± 2.11a
	*ACE2*	1.11 ± 0.54a	0.91 ± 0.13a	1.38 ± 0.98a	1.77 ± 1.06a
	*AT*_1_ *R*	0.98 ± 0.53a	0.79 ± 0.34a	2.03 ± 3.08a	1.23 ± 0.59a
	*Mas1*	0.98 ± 0.57a	0.98 ± 0.64a	1.51 ± 0.77a	0.78 ± 0.22a
thoracic aorta	*ACE*	3.01 ± 3.46a	1.23 ± 1.90a	0.67 ± 0.38a	5.04 ± 4.12a
	*ACE2*	0.97 ± 0.58a	0.91 ± 0.51a	0.29 ± 0.08a	0.75 ± 0.38a
	*AT*_1_ *R*	1.22 ± 0.78a	0.83 ± 0.38a	0.96 ± 0.47a	0.95 ± 0.53a
	*Mas1*	1.24 ± 0.74a	0.91 ± 0.44a	0.78 ± 0.42a	1.29 ± 0.83a
abdominal aorta	*ACE*	1.58 ± 1.44a	1.08 ± 0.44a	2.19 ± 1.56a	1.12 ± 1.24a
	*ACE2*	0.24 ± 0.19a	ND	0.14 ± 0.11a	0.43 ± 0.32a
	*AT*_1_ *R*	0.98 ± 0.32a	1.60 ± 1.25a	0.97 ± 0.64a	1.27 ± 1.10a
	*Mas1*	1.17 ± 0.71a	1.13 ± 0.58a	0.90 ± 0.52a	0.64 ± 0.22a

Control, *n* = 9. CEF, ceftriaxone group, *n* = 9. HFD, high-fat diet group, *n* = 8, CEF + HFD, ceftriaxone and high-fat diet group, *n* = 9. No common letter in each row means *p* < 0.05. ND, not detected. The changed genes were emphasized by bold face font.

## Data Availability

All data generated or analyzed during this study are included in this published article. If the raw data must be uploaded, the author can provide it.

## Supplementary Materials

The following supporting information can be downloaded at: https://www.mdpi.com/article/10.3390/nu15122661/s1, Table S1: Components of regular diet; Table S2: Components of high-fat diet; Table S3: The thermal cycling protocol of reverse transcript PCR; Table S4: The thermal cycling protocol of qPCR; Table S5: The primer sequences.
