# Supplementary material for: Gut Microbiota Perturbation in Early Life Could Influence Pediatric Blood Pressure Regulation in a Sex-Dependent Manner in Juvenile Rats
Source: Nutrients. 2023 Jun 7;15(12):2661. doi: 10.3390/nu15122661 (PMC10304250; doi:10.3390/nu15122661)
Supplement: Supplementary file 1 [file nutrients-15-02661-s001.zip › nutrients-2401526-supplementary.pdf]

Table S1. Components of regular diet.

Component	Content (%)
Crude Protein	≥18.0
Ether Extract	≥4.0
Crude Fiber	≤5.0
Crude Ash	≤8.0
Phosphorus	0.6~1.2
Calcium	1.0~1.8

Table S2. Components of high-fat diet.

Component	Content (%)	kcal%
Protein	26.2	20
Carbohydrate	26.3	20
Fat	34.9	60

Table S3. The thermal cycling protocol of reverse transcript PCR.

Step	Parameter
Priming	5 min at 25 °C
Reverse transcription	20 min at 46 °C
RT inactivation	1 min at 95 °C
Final step	Hold at 4 °C

Table S4. The thermal cycling protocol of qPCR.

Step	Parameter
Polymerase activation and DNA denaturation	30 s at 98 °C
Denaturation	15 s at 98 °C
	10 s at 60 °C (for *Ren*, *ACE*, *Mas1*)
Extension	20 s at 60 °C (for *GAPDH*, *AGT*, *AT1R*)
	30 s at 60 °C (for *ACE2*)
Plate read	-
Cycles	40
Melt curve analysis	65–95 °C (0.5 °C increments)

Table S5. The primer sequences.

Gene	Forward	Reverse
GAPDH	5-GCCGCATCTTCTTGTGCAG-3	5-ATGAAGGGGTCGTTGATGGC-3
AGT	5-GAGCCAACCTTTGAGCCTGT-3	5-CGGAAACCCATGAAGTTGGC-3
Ren	5-AAGATGTGGTAACTGTGGGTGG-3	5-CTTGGCCAGCATGAAGGGTAT-3
ACE	5-TCTGTCACTGGAGCCTGATCT-3	5-AGCTCTTCCACACCCAAAGC-3
ACE2	5-TGACAATTGTTGGAACGCTGC-3	5-CAACGATCTCCCGCTTCATCT-3
AT1R	5-TTTCTTACCGGCCTTCGGAT-3	5-GCCATTTAGTCAGAGGCTGC-3
Mas1	5-GGTCTTCACTCCGCTCATGT-3	5-AGCTTCGAAGAATGGGAGGC-3
